# Tissue-engineered vocal fold replacement in swine: Methods for functional and structural analysis

**DOI:** 10.1371/journal.pone.0284135

**Published:** 2023-04-21

**Authors:** Patrick Schlegel, Kenneth Yan, Sreenivasa Upadhyaya, Wim Buyens, Kirsten Wong, Anthony Chen, Kym F. Faull, Yazeed Al-Hiyari, Jennifer Long

**Affiliations:** 1 Department of Head and Neck Surgery, David Geffen School of Medicine at the University of California-Los Angeles, Los Angeles, California, United States of America; 2 Department of Otolaryngology/Head and Neck Surgery, Rutgers New Jersey Medical School, Newark, New Jersey, United States of America; 3 Department of Computer Science, Katholieke Universiteit Leuven, Leuven, Belgium; 4 SoundTalks N.V, Leuven, Belgium; 5 Department of Medicine, David Geffen School of Medicine at the University of California-Los Angeles, Los Angeles, California, United States of America; 6 Department of Psychiatry & Biobehavioral Sciences, Pasarow Mass Spectrometry Laboratory, Jane & Terry Semel Institute of Neuroscience and Human Behavior, David Geffen School of Medicine at the University of California-Los Angeles, Los Angeles, California, United States of America; 7 Surgery and Perioperative Careline, Greater Los Angeles VA Healthcare System, Los Angeles, California, United States of America; University of Vermont College of Medicine, UNITED STATES

## Abstract

We have developed a cell-based outer vocal fold replacement (COVR) as a potential therapy to improve voice quality after vocal fold (VF) injury, radiation, or tumor resection. The COVR consists of multipotent human adipose-derived stem cells (hASC) embedded within a three-dimensional fibrin scaffold that resembles vocal fold epithelium and lamina propria layers. Previous work has shown improved wound healing in rabbit studies. In this pilot study in pigs, we sought to develop methods for large animal implantation and phonatory assessment. Feasibility, safety, and structural and functional outcomes of the COVR implant are described. Of eight pigs studied, six animals underwent COVR implantation with harvest between 2 weeks and 6 months. Recovery of laryngeal tissue structure was assessed by vibratory and histologic analyses. Recovery of voice function was assessed by investigating acoustic parameters that were derived specifically for pigs. Results showed improved lamina propria qualities relative to an injured control animal at 6 months. Acoustic parameters reflected voice worsening immediately after surgery as expected; acoustics displayed clear voice recovery in the animal followed for 6 months after COVR. These methods form the basis for a larger-scale long-term pre-clinical safety and efficacy study.

## Introduction

The vocal folds (VF) are a three-layered structure composed of a stratified squamous epithelium, lamina propria, and muscle, of which lamina propria vibration produces the sound source of voicing [1,2]. Unlike other stratified squamous epithelia in the human body, the VFs withstand cyclical tensile, shear, and impact stresses, the effect of which is to require continual repair [3]. Unfortunately, vocal fold scarring formed as a result of trauma, surgery, or inflammation impairs the healing process, leading to abnormal collagen deposition and extracellular matrix (ECM) derangement within the lamina propria, thus impairing phonation. Currently, there is no optimal treatment for severe VF scarring, leaving affected patients with hoarse voices, vocal fatigue, and impaired professional careers and social interactions.

The lamina propria is a complex connective tissue comprising organized collagen and elastin fibers with interspersed hyaluronic acid. Regenerative medicine has the potential to replace or repair this structure using synthetic polymer scaffolds, cell transplantation and other state-of-the-art biomaterials to restore the vocal fold’s capability for phonation [4–7]. Specifically, recent research into the use of mesenchymal stem cells (MSCs) to repair scarring has been promising [8,9]. A recent phase I/II clinical trial in 16 patients examined the therapeutic efficacy of autologous MSC injection, demonstrating objective improvements in VF vibration and phonation threshold pressure in two-thirds of the patients [10].

A more extensive approach may be needed for those unresponsive to simple cell injections, or for primary VF reconstruction at the time of cancer resection. Three-dimensional mucosal VF replacements have been developed to serve this need [11–13]. We have employed human adipose-derived MSCs (hASCs) within a fibrin matrix to create a Cell-based Outer Vocal fold Replacement (COVR). The COVR is constructed *in vitro*, using epidermal growth factor and an air-liquid interface to promote a bilayered organization of epithelial and mesenchymal cells within the scaffold [12]. When applied in rabbits, the COVR implant incorporated into the larynx without excessive scar formation and produced excellent vibration in an excised larynx system [14]. Functional results were superior to VF auto-transplantation, indicating that the regenerative properties of the COVR were more beneficial than simply supplying mature native VF [2,14].

Whether the COVR acts as a permanent graft or if it serves as a scaffold to allow migration of native cells and facilitate wound healing remains an ongoing question. In our previous work in rabbit models, immunohistochemical staining and in-situ hybridization studies demonstrated persistence of some implanted hASCs for 4–6 weeks [15,16]. However, host cells were also present in the area. These observations suggest that a hybrid lamina propria forms, with some implanted cells persisting while host cells also migrate into the region. Limitations of these previous studies include the short follow-up time and limited functional assessment that could be performed in a non-vocal mammal (rabbits). Thus, it remains an ongoing question whether the COVR implant itself is responsible for phonation, or whether the COVR serves to manipulate the host wound healing response.

The goal of this study was to develop methods for COVR implantation in a swine model to ultimately enable functional voice assessment. The pig larynx exhibits a lamina propria structure similar to the human larynx [17]. When compared to other species, pig vocal folds also have a non-linear stress-strain relationship similar to humans, and a high range of phonation frequencies, thereby making it a favorable model for pre-clinical studies [18]. Pigs have also been used for implantation of composite laryngeal framework replacements including cartilage and muscle [19–21]. Accordingly, we hope that by first demonstrating the feasibility, safety and efficacy of the vibratory COVR implant in a pig model, it may next be translated for use in humans. For this study, we developed the surgical procedure and perioperative protocol for COVR implantation in Yucatan mini-pigs. Methods were also developed to assess vocal fold structure and phonatory function. Assessing efficacy was not the goal at this stage; rather, the intention was to refine methods before commencing a larger controlled study.

From a structural standpoint, we performed histology and multiphoton microscopy to assess the ability of the COVR to recapitulate the native VF lamina propria. We additionally quantified the recoveries of collagen and elastin content in the early post-implant period by measuring their unique amino acids, hydroxyproline (4HOP) and desmosine (D), respectively. From a functional standpoint, we applied an in-vivo phonation method to demonstrate vibrational recovery, and performed acoustic analyses of spontaneous vocalizations to quantify vocal recovery in COVR-implanted pigs. Objective assessment of functional recovery in pig voices is challenging as there are no measures specifically designed for this task [22]. In a previous study we investigated various measures that had been used elsewhere to differentiate aperiodic pig voice calls [22–25]. In the current study, we use the two measures found in our previous work that best reflect the effects of surgery on pig voice (50% energy spectrum quantile and spectral flux). Finally, we investigated a set of 107 acoustic measures that are used in commercial swine health evaluations, which were narrowed to four relevant measures that best reflected the effects of vocal fold surgery.

## Materials and methods

### Adipose-derived mesenchymal stromal cells and COVR development

COVRs were constructed two weeks prior to implantation as previously described [13]. Briefly, hASCs (ATCC) were expanded in culture for 2–5 passages until use. Bovine fibrinogen, recombinant human thrombin in calcium chloride, and an hASC suspension at 6 x 10^6 cells/mL were mixed at a 4:1:1 ratio in 12-mm Transwell (Corning) inserts for 12-well culture plates. Inserts contained a porous polycarbonate membrane (0.4 μm pore size) at the base, to allow for culture medium delivery from the base only. Gelation was achieved within 30 minutes of incubation at 37°C. hASC-fibrin gels were maintained with culture medium containing 10% fetal bovine serum and 10 ng/mL recombinant human epidermal growth factor for 2 weeks. Media was exchanged every 2–3 days until time of implantation. The resultant neotissue constructs were cylindrical, 12mm in diameter, and 2–3 mm in height, containing approximately 600,000 cells. At the time of implantation, gels were harvested by separating the Transwell membrane and the gel from the insert with a scalpel and peeling the membrane from the gel while maintaining its orientation. hASCs destined for injection in the vocal fold were harvested immediately prior to surgery. 600,000 cells were prepared in a total volume of 0.4 mL of PBS and stored in a capped syringe on ice until ready for injection.

### Laryngeal surgery, COVR implantation, and immunosuppression

This study was performed in accordance with the PHS Policy on Humane Care and Use of Laboratory Animals, the NIH Guide for the Care and Use of Laboratory Animals, and the Animal Welfare Act (7 U.S.C. et seq.) All animal activities were done in accordance with the University of California-Los Angeles Institutional Animal Care and Use Committee. Eight Yucatan mini pigs (four males and four females) were employed (Table 1). Animals 1–4 were planned for a unilateral implant with a 6-month harvest timepoint to assess long-term recovery of vocal fold structure and voice function. Animals 5–8 were planned for a bilateral implant and early harvest (between 2–6 weeks) for molecular analysis during the early wound healing phase.

**Table 1:** Chronology of pigs used during pilot studies.

Pig #/ sex	Age, weight at surgery	Treatment	Immuno- suppression	Harvest timepoint	Outcome measures	Cause of death
1. Male	18 weeks 20 kg	Endoscopic left VF resection, hASC injection	Cyclosporine, Prednisolone	6 months	1,2	Reached timepoint
2. Female	17 weeks 26 kg	Endoscopic right VF resection alone	Prednisolone	6 months	1,2	Reached timepoint
3. Male	12 weeks 13 kg	Right COVR	Cyclosporine, Prednisolone	40 days	3	Meningitis (at day 40)
4. Female	19 weeks 25 kg	Right COVR	Prednisolone	6 months	1,2,3,4	Reached timepoint
5. Female	23 weeks 32 kg	Bilateral COVR	Prednisolone	4 weeks	5	Reached timepoint
6. Male	30 weeks 33 kg	Bilateral COVR	Prednisolone	2 weeks	5	Reached timepoint
7. Female	20 weeks 24 kg	Bilateral COVR	Prednisolone	6 weeks	5	Reached timepoint
8. Male	34 weeks 35 kg	Right COVR	None	6 weeks	3,5	Reached timepoint

Outcome measures: 1 Histology, 2 Second harmonic generation (SHG) microscopy, 3 Acoustic analysis, 4 Terminal phonation, 5 Single cell RNA sequencing.

Each pig was sedated with intramuscular Telazol (tiletamine) to enable intravenous catheter placement in the tail vein. Anesthesia was then induced with inhaled isoflurane and intravenous Propofol (diprivan). A size 6–0 endotracheal tube was placed by an experienced veterinary technician.

Initially, endoscopic implantation was planned in order to simulate the expected human surgery. This approach was attempted in the first two pigs. A standard Dedo laryngoscope was found to be too large for the pig’s narrow pharynx. Instead, a Hollinger anterior commissure laryngoscope was inserted successfully and suspended. A 0-degree Hopkins rod telescope provided visualization during unilateral endoscopic vocal fold stripping. The vocal fold mucosal resection was performed by grasping one membranous VF mucosa with cupped forceps and resecting down to muscle. COVR implantation was attempted, but the surgical access was found to be inadequate for manipulation and endoscopic suturing. With COVR implantation found to be impossible at that time, alternate treatments were undertaken. Pig 1 underwent injection of 600,000 hASCs in PBS, directly into the vocal fold wound bed, to assess the impact of cell injection therapy at an equivalent dose to the COVR. Pig 2 underwent injury alone, with injection of PBS alone into the vocal fold wound bed.

**Fig 1:**
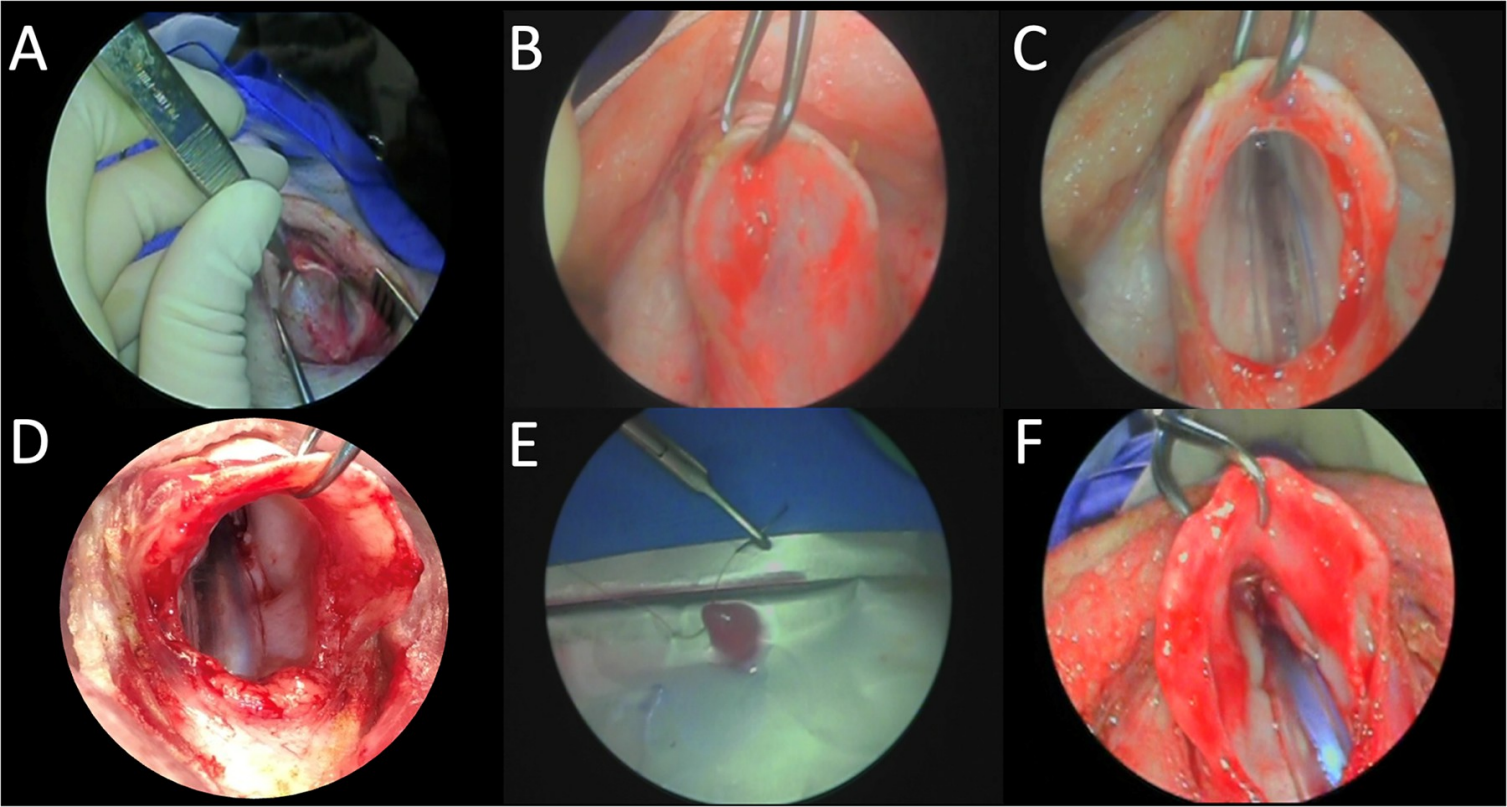
Transcervical COVR implantation in a Yucatan mini-pig. A) Retracting the vertical midline incision allows B) elevation of thyroid cartilage. C) Incision of thyrohyoid membrane exposes endotracheal tube. D) View of right vocal fold after resection of epithelium and lamina propria. E) Suture passage through COVR implant. F) Bilateral vocal fold COVR implants in place.

For pigs 3–8, a transcervical surgical implantation method was developed to enable reliable vocal fold injury and implantation (Fig 1). A midline neck incision with a sub-hyoid pharyngotomy approach to the larynx offered excellent surgical access to both vocal folds while protecting the airway with endotracheal intubation. Specifically, a vertical midline neck incision was made to expose the laryngeal cartilage. The endolarynx was then accessed by incising the thyrohyoid membrane for a midline pharyngotomy approach. This enabled direct visualization and manipulation of both vocal folds, with the endotracheal tube positioned posteriorly. Because intralaryngeal access was sufficient, laryngofissure was not needed.

**Fig 2:**
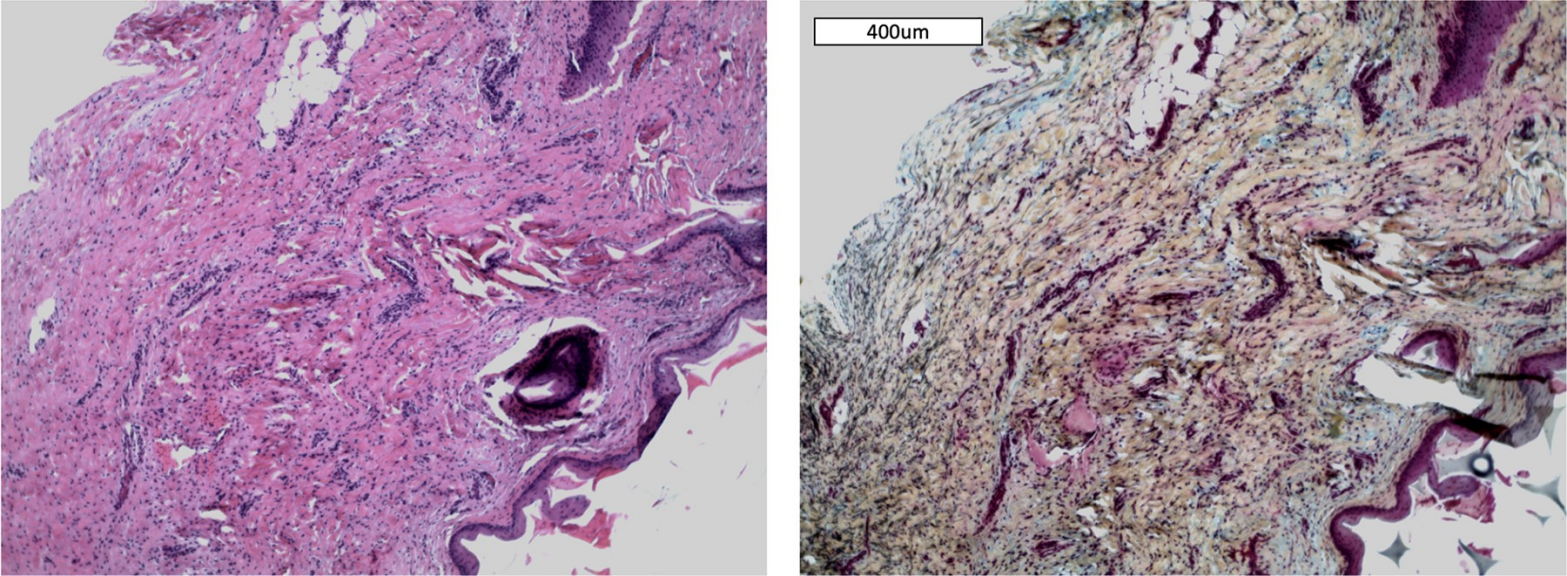
Excised normal VF collected during implant surgery, 10X. Movat pentachrome stain shows elastic fibers (black), collagen (yellow) and glycosaminoglycans (blue). Epithelium appears at bottom right corner.

The membranous cover layer was then resected from one or both true vocal folds by sharp dissection (Fig 2). Resection extended from immediately anterior to the arytenoid cartilage to immediately posterior to the anterior commissure, leaving 1–2 mm of normal vocal fold at each border. Mucosa was removed down to thyroarytenoid muscle, equivalent to a European Laryngological Society type 2 cordectomy. Cell-based Outer Vocal Fold Replacements were secured onto the defect with anterior and posterior 5–0 plain gut sutures. The pharyngotomy incision was closed with airtight 3–0 Vicryl sutures, and the neck was closed in a layered fashion. The endotracheal tube was removed gently after the animal awoke from anesthesia. All animals tolerated surgery well, returned to ambulatory state within minutes after emerging from anesthesia, and proceeded to oral diet the same day.

Transplant immunosuppression with cyclosporine and prednisolone was initially undertaken for these pigs receiving human cells, to prevent immune rejection and any laryngeal reaction. Pig 1 was treated with this protocol after receiving cell injection. Pig 2 did not receive human cells, and in consultation with the veterinarian cyclosporine was withheld to avoid drug-related comorbidities. Prednisone was continued in pig 2 for consistency with other animals because of its potential influence on vocal fold wound healing. Pig 3 did receive both cyclosporine and prednisolone but developed a fatal meningitic infection under this immunosuppressant protocol. Therefore, pigs 4–7 were given milder immunosuppression with prednisolone alone. Pig 7 subsequently fell ill due to an endemic porcine bacterial infection but survived to planned euthanasia. To avoid further infectious complications, pig 8 was treated with only 3 days of perioperative dexamethasone without long-term immunosuppression or corticosteroids. Table 1 summarizes the animal treatments.

### In-vivo phonation

Pig 4 underwent induced phonation *in-vivo* with high-speed laryngoscopy at 6 months after COVR implantation, immediately prior to euthanasia and laryngeal harvest. The induced phonation method was adapted from similar procedures described elsewhere for canines [26,27]. The animal was anesthetized, intubated, and positioned supine with neck extension. A midline vertical incision over the larynx was performed and lateral retraction exposed the larynx. A tracheostomy tube was inserted for ventilation, and the oral endotracheal tube was removed. A second tube to supply airflow for phonation to the larynx was inserted from inferior. Vagus nerves were identified bilaterally and hook electrodes placed for nerve stimulation. The larynx was divided superiorly from the pharynx and the supraglottic structures resected to allow a clear superior view of the vocal folds.

After suspension of the larynx in this manner, phonation was induced by simultaneously stimulating both vagus nerves while supplying humidified airflow upwards through the glottis. A high-speed digital videocamera (Phantom v210; Vision Research Inc., Wayne, NJ) recorded the laryngeal vibration at 5,000 frames per second with a resolution of 512 × 800 pixels. A segment of video is included in the supporting information (S1 Video). Vocal fold appearance, glottic closure, and mucosal wave symmetry were assessed qualitatively.

### Tissue preparation and microscopy

Animals were euthanized at the timepoints indicated in Table 1, and larynges were excised. Vocal folds from pigs 1, 2, and 4 were prepared for microscopy. Both vocal folds were dissected free from the laryngeal framework, and a mid-membranous segment encompassing the true and false glottic folds was oriented in a standard tissue cassette for formalin fixation and paraffin embedding. Sections were made in the coronal plane to show the entire thickness of the VF from thyroarytenoid muscle to epithelium (see Results). Histologic stains included hematoxylin and eosin, and Movat’s pentachrome.

Nonlinear laser scanning microscopy with second harmonic imaging was used to detect collagen and elastic fibers. Unstained 5 μm sections were imaged using a Leica SP8 MP-DIVE Fluorescence Lifetime Imaging Microscope. Excitation and collection wavelengths were optimized for this tissue as follows: two-photon laser wavelength of 830nm and power setting of 500mW (25% of maximum); collagen imaging by second harmonic generation imaging in reflected imaging mode with detection wavelengths of 405–425 nm; elastin imaging by transmitted autofluorescence with detection wavelengths of 522–532 nm. Images were collected using a 40x objective lens with water immersion. The entire section was scanned to create a mosaic of the tissue section, and 5 z-level images were projected. Lamina propria was manually selected for image analysis by drawing a region of interest that excluded epithelium and thyroarytenoid muscle. A smaller area selection is presented in Results to show fiber detail. For consistency, collagen is displayed as green and elastin as magenta.

### Image-based analysis of fiber directionality and distribution

Second harmonic and autofluorescence microscopic images were collected from pig 1 (VF resection and hASC injection), pig 2 (VF resection without reconstruction) and pig 4 (VF resection and COVR implant) at the 6-month timepoint as described above. Using these images of the entire lamina propria, co-alignment of collagen and elastic fibers (i.e. if both types of fibers were oriented in the same direction) and uniformity of fiber orientations were algorithmically assessed. Fiber alignment difference was measured in degrees deviation between average orientation of collagen and elastic fibers. Uniformity of orientation is measured as dispersion of the angular orientation distribution, such that higher dispersion indicates that the different collagen or elastin fibers within a sample are oriented more randomly.

**Fig 3:**
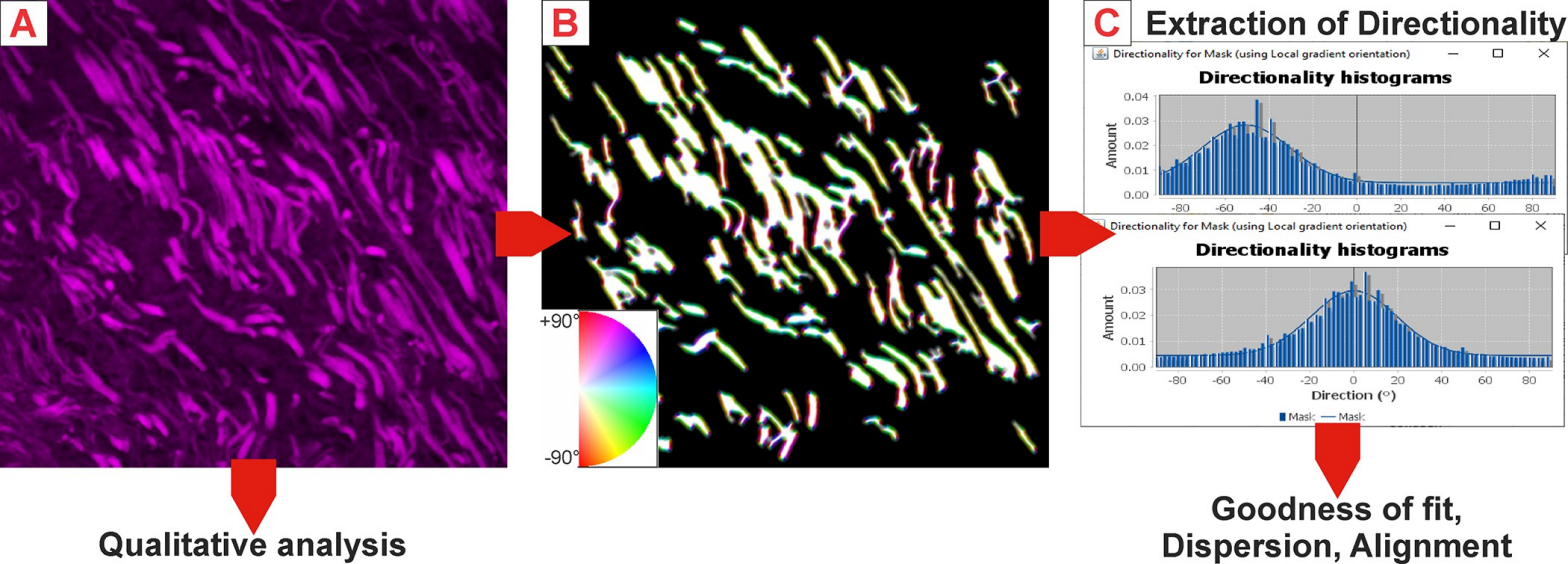
Brief illustration of image fiber analysis covering (A) qualitative analysis based on raw images, (B) computation of a mask, mean filtering and directionality detection with color-coded pixel orientations and (C) normal distribution fits and parameter extraction.

Single-channel mosaic images were exported for quantitative fiber analysis using ImageJ (FIJI) (version: 1.51). For each channel (collagen and elastin), pixel noise was reduced using a median filter (2-pixel width). Intensity was normalized across images. Additionally, for the unoperated control, a saturated pixel threshold of 4% was applied, brightening the image. This compensated for low overall light intensity in that image, which would have introduced artificial differences in the evaluation. Otsu auto-thresholding was used (ignoring black areas), generating a binary mask for each image. Small artifacts were removed using the “analyze particles” function with a threshold of 50 μm^2^. After mean filtering (3-pixel width) of the resulting images, fiber directionality (orientation of detected fiber fragments / pixels between 0° and 180°) was determined using “Local gradient orientation”. During directionality detection, each pixel was assigned an orientation based on neighboring structures. By summating the number of pixels for each direction, a “directionality histogram” for each image was calculated, denoting frequency of all orientations. The image preprocessing and data extraction process is illustrated in Fig 3.

A normal curve was fitted to directionality histograms, and alignment (position of the peak of the fitted curve) was calculated. The distribution was then centered at 0° and re-fitted to avoid artifacts outside the peak area (see Fig 3C). Goodness of fit (GoF, range from 0 to 1) was calculated for the centered distribution. Dispersion (a measure of distribution width) was extracted from the fitted curve.

### Amino acid quantitation

Desmosine (D) and 4-hydroxyproline (4HOP) were quantified by a liquid chromatographic/tandem mass spectrometric method in one normal pig and pigs 5, 6 and 7 who received the COVR implant. Briefly, upon collection, vocal folds were immediately frozen on dry ice, and then lyophilized in microcentrifuge tubes. The lyophilized samples were then homogenized in water in a bead beater using stainless steel beads, followed by centrifugation. Precipitates were then treated with 6N HCl (120°C, 16h). The samples were dried in a vacuum centrifuge, and the residues redissolved in water. Isotopically labeled internal standards (^2^H_4_-D and ^2^H_3_-4HOP) were added to an aliquot of the resulting solution. The samples were thoroughly dried again in a vacuum centrifuge and then treated with n-butanoic HCl (60°C, 1h). The reaction mixtures were dried again in a vacuum centrifuge, residues redissolved in water and aliquots injected into a high-pressure reversed phase liquid chromatography column equilibrated in water/formic acid. The columns were then eluted with increasing concentrations of acetonitrile/formic acid. The effluent from the column was directed to an electrospray ionization source connected to a hybrid mass spectrometer (Thermo LTQ XL) operating in the linear ion trap positive mode where preselected parent ions were fragmented using collisionally activated dissociation. The areas of the chromatographic peaks corresponding to parent-to-fragment ion transitions were recorded and measured with manufacturer-supplied software (Xcalibur™ Qual Browser). Each batch of samples was quantified against a series of standards containing the same internal standards and increasing amounts of both D and 4HOP. All runs were performed in duplicate.

### Acoustic recording and labeling

Three pigs that underwent unilateral COVR implant were studied for acoustic analysis (pigs 3, 4, and 8). Bilateral implants were excluded from the acoustic studies because the bilateral injury worsened initial voice quality, which could not be directly compared with the unilateral case. The recording setup for spontaneous pig phonation was described in our previous study, and pigs 3, 4 and 8 are identical to pigs reported as numbers 1, 2 and 3 in that work [22]. However, for this work, none of the previously investigated acoustic data was re-used; in the previous work only a small sample of squeals was analyzed to assess changes in parameters. Pigs 1–4 were housed singly, and spontaneous vocalizations were recorded for 48-hour periods within their home enclosure. Pigs 5–8 were group-housed to satisfy their social needs, and were moved to the solitary enclosure for 6-hour periods for acoustic recording. Consequently, fewer squeals were collected for those later pigs. All pigs were free to roam around the enclosure, eat and sleep during the recording time. A digital audio recorder (H4n Pro, 140 dB SPL, minimum sensitivity -12dB, 16 bit, 44100 Hz sampling rate, stereo) was secured at the entrance of the 7.43 m^2^ pig enclosure, 0.75 m above ground level.

**Fig 4:**
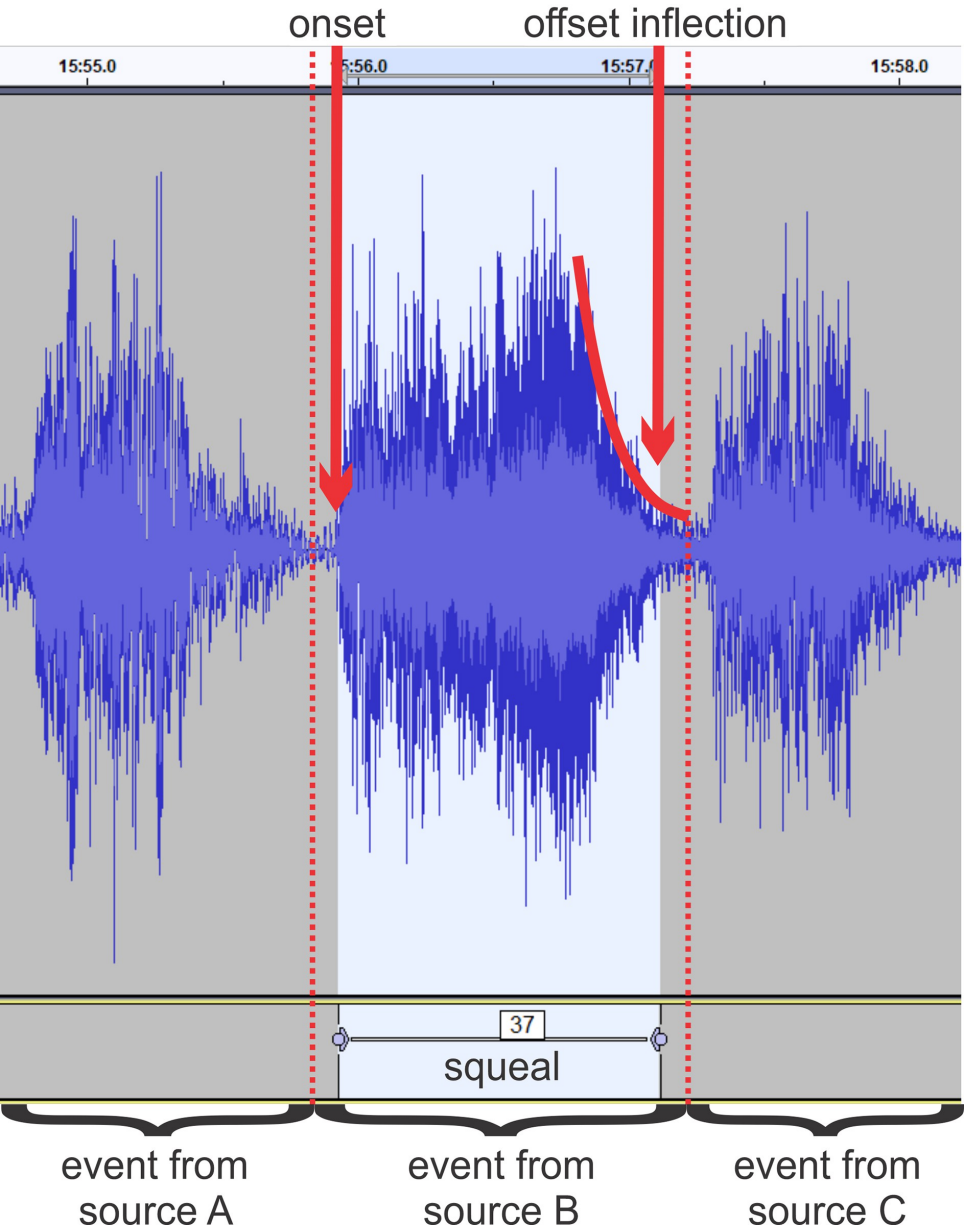
Labeling of a squeal within one event in a mixed file.

Raw audio recordings were processed into 85 “mixed files” each containing about two hours of acoustic events in random order. To ensure double-blind labelling, raw data was separated into multiple short “event” sections using a moving average volume threshold of 500. Additional acoustic events recorded from seven normal pigs were then introduced into the mixed files to reduce rater bias due to the otherwise low number of “healthy squeal” recordings. Three raters then listened to the files and marked squeal-events based on the following criteria: subjective high pitch, minimum duration of 0.5 seconds without overlapping vocalizations or noise. Start positions were set immediately before a sharp amplitude increase (i.e. at onset). End positions were set as the phonation fades away, at a subjective turning point between exponential and linear amplitude decline (at offset inflection, see Fig 4). Using this approach, 5534 squeals from three pigs were extracted [28] for analysis using the parameters described in the next section (see Table 2).

**Table 2:** Number of squeals included in analysis pre- and post-surgery, number of post-surgery recording sessions per pig and type of analysis performed for each pig.

Pig	Squeals pre surgery	Squeals post-surgery	Recording sessions post-surgery	Analysis method
3	688	2021	4	Evaluation A&B
4	423	2300	17	Evaluation A&B
8	65	37	2	Only evaluation A

### Acoustic parameter analysis

Two separate analysis pathways were performed independently. Squeals from all 3 pigs (pigs 3, 4, and 8) underwent evaluation A, based on findings in our previous work [22]. A second, more extensive analysis (evaluation B) was conducted on squeals from the two pigs with substantially more acoustic recordings (pigs 3 and 4, as noted in Table 2).

**Fig 5:**
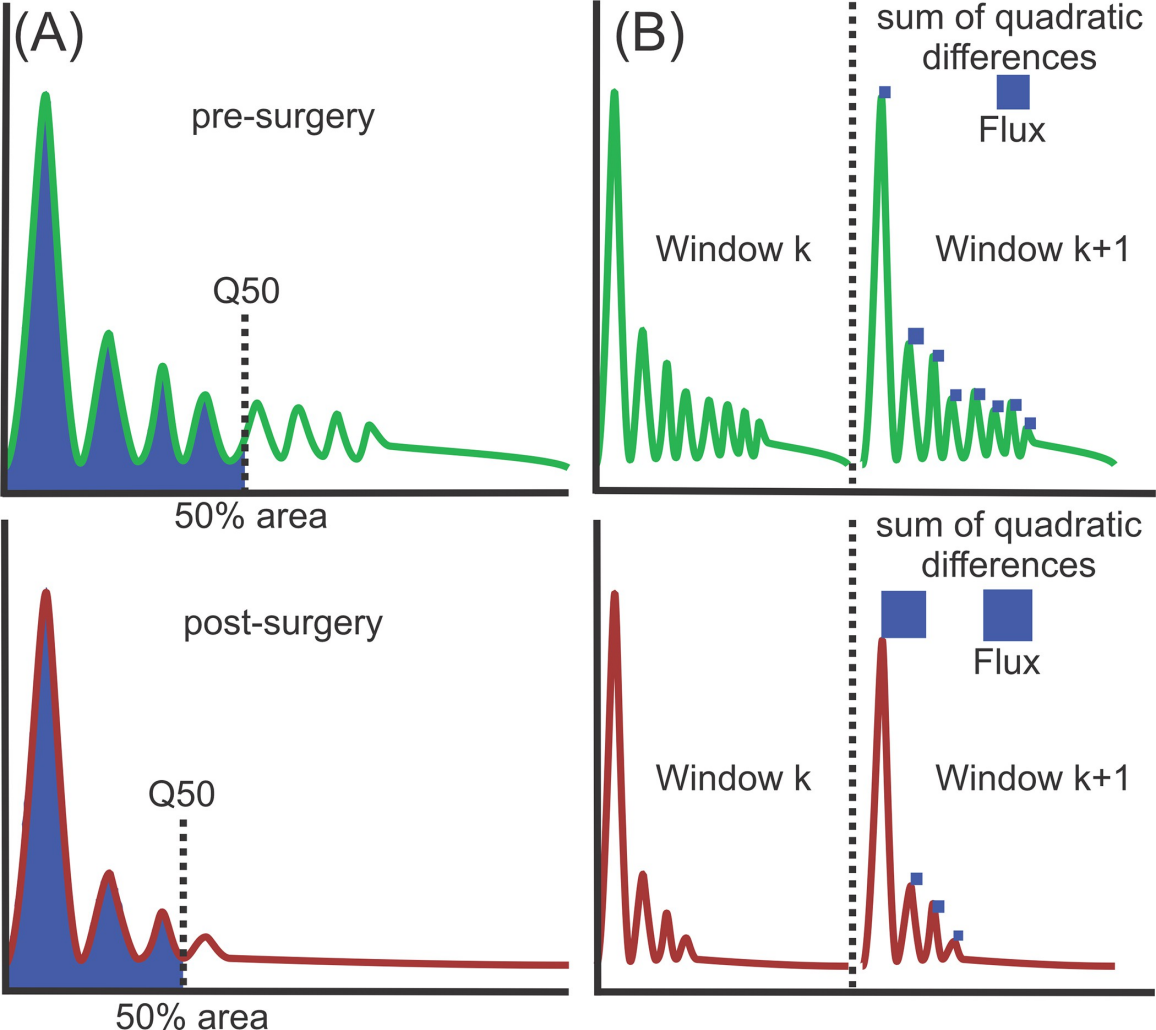
Illustration of calculation of (A) Q50 and (B) Flux. More energy in higher energy leads to higher Q50. Higher distortions in especially the lower frequencies between neighboring energy spectrum windows lead to higher Flux.

For evaluation A, two parameters were calculated for all squeals: the 50% energy spectrum quantile (Q50) and Spectral Flux (Flux1). Q50 describes the frequency that divides the energy spectrum of a signal into two intervals of equal energy; a higher Q50 indicates a greater contribution of higher frequencies to the energy spectrum. This parameter captures the loss of high frequencies found in pig squeals after surgery. Flux1 represents the average difference in energy between all neighboring energy spectral windows (see Fig 5). It reflects an increase in phonation instabilities after surgery. For consistency, Matlab implementations previously published [22] were used for the calculation of Q50 and Flux1 in this work and Matlab (version 2021b) was used for analysis.

Evaluation B was undertaken to validate the results of evaluation A, and to expand to a broader spectrum of potentially useful parameters. 107 different acoustic measures were investigated. This number was reduced to 36 features using a Gaussian mixture model. For this process, pre- and post- surgery squeals for each of the two pigs were compared. The distribution of the features for the pre- and post- surgery groups were modeled as Gaussian distributions, and the overlap of the distributions was calculated. A small overlap between the distributions implies greater differentiation for the feature between the pre- and post-surgery states.

The 4 features with the most relevant trends–Flux2, Spread, P60 and LPC8 –are discussed in this work. Flux2 (similar to Flux1) is a measure of spectral change but calculated using a different algorithm [29]. Spread is a Mel spectrum-based measure that represents the average deviation of the frequency components in the Mel spectrum of the event around the centroid [30]. It is expected to increase with signal noise. Similar to Q50, P60 represents the frequency component in the Mel spectrum at which 60% of the total energy is reached [31]. Lastly, LPC8 is the 8th linear predictive coding coefficient out of a total of 16. Linear Predictive Coding is typically used to analyze a speech signal by estimating the formants produced by the vocal tract; major changes in the vocal tract typically result in changes in these coefficients [32]. To illustrate low correlation between these four parameters, bias corrected distance correlation as defined by Székely and Rizzo [33] was measured.

### Statistical analysis of acoustic parameters

**Fig 6:**
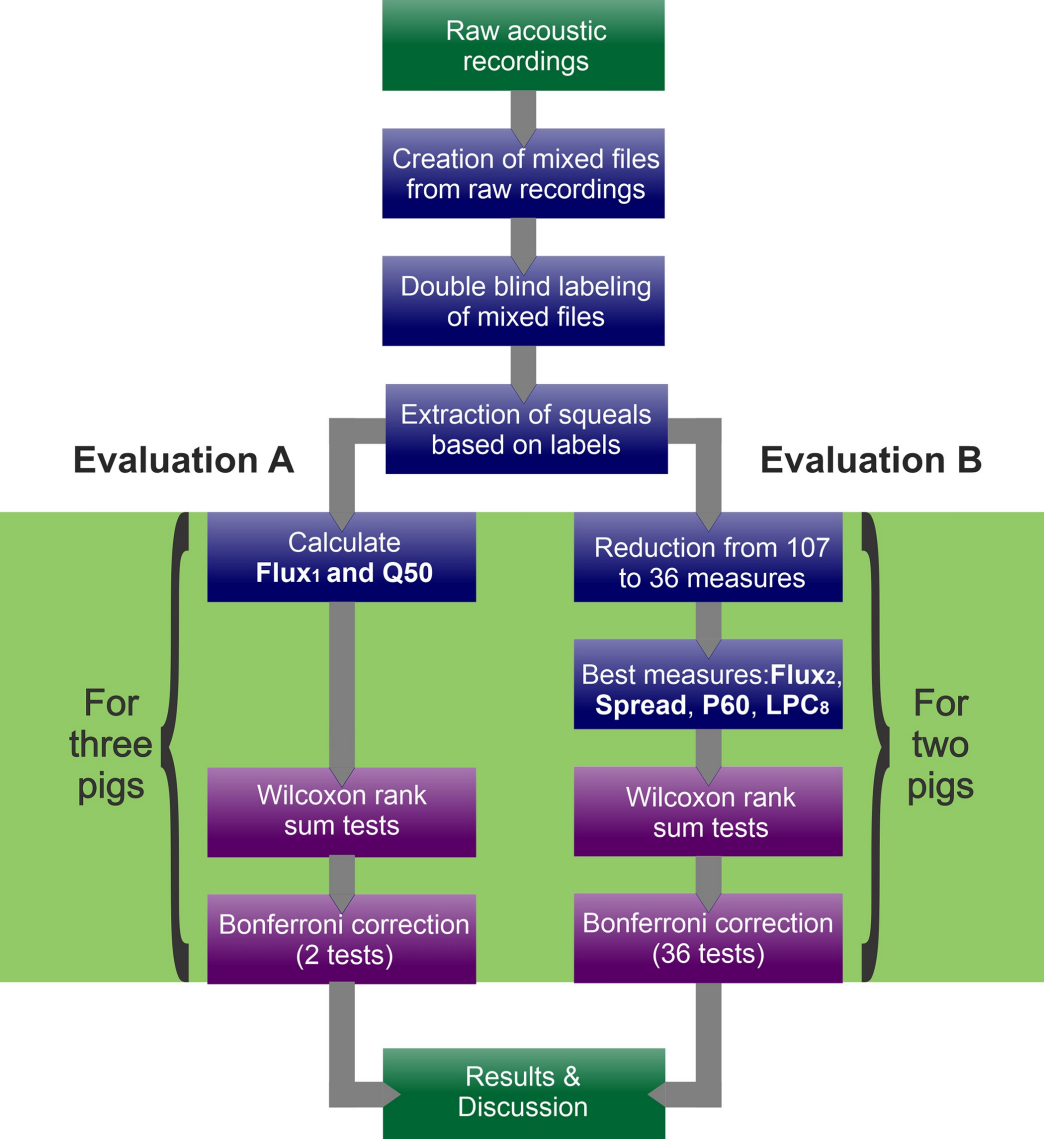
Summary of squeal analysis. In each pathway (evaluation A and B), preoperative and early post-operative (0–14 days) squeals were compared. Additionally, pig 4 had comparison of early versus late post-operative squeals.

For pre- and post-surgery comparisons in pigs 3, 4 and 8, equal numbers of randomly chosen samples from all dates up to 33 days before surgery were compared with samples recorded up to 2 weeks post-surgery. In this way, overrepresentation of single dates was avoided. Pre- and post-surgery data were compared using one-sided Wilcoxon rank sum tests for all parameters found in evaluation A and the four final features in evaluation B. Directions of tests along with parameter names, units, short descriptions and sources are given in S1 Table. p-values for multiple testing were corrected using Bonferroni correction at 5% for two tests (evaluation A) and 36 tests (evaluation B). This approach was chosen over a more liberal false discovery rate correction as the latter would have required calculation of p-values for all 36 features in evaluation B [34]. However, p-values could not be calculated for all of these features because a direction for testing could not always be determined based on the parameter properties. Correction was applied separately for all pigs. Additionally, long-term trends in pig 4 were assessed by comparing 50–100 and 150+ days after surgery to the early post-surgery parameters for this animal (0–14 days). In these cases, test directions were inversed to test for parameter improvement. A summary of the entire analysis process is given in Fig 6.

## Results

### Surgical feasibility and safety

The first four pigs in the study (Pig 1 to 4) underwent vocal fold resection with the plan for harvest at 6 months. In pigs 1 and 2, COVR implantation was attempted but was not possible due to difficult pig anatomy which limited endoscopic access of surgical instruments to the larynx. Therefore, pig 1 had endoscopic injection of human adipose stem cells (hASCs), while pig 2 was maintained as an injured control. Pigs 3 and 4 underwent successful transcervical surgery for unilateral VF replacement. Pigs 5 through 8 underwent successful unilateral or bilateral open vocal fold resection and COVR implantation. Vocal folds were harvested from these pigs at 2, 4 and 6 weeks. These vocal folds were subsequently used for genomic and proteomic analysis, which is ongoing. They are included in this series because they contributed to the development of methods and acoustic analysis.

Because the COVR contains human cells, initial porcine implantation experiments were performed with transplant immunosuppression of cyclosporine and prednisolone. However, Pig 3 developed a fatal meningitic infection under this immunosuppressant protocol. Therefore, Pigs 4–7 were given milder immunosuppression with prednisolone alone. Consideration was given to eliminating prednisolone as well, but the decision was made to continue prednisolone to prevent excess laryngeal edema. Pig 7 subsequently fell ill due to Streptococcus suis (an endemic porcine bacterial infection), but survived to its planned harvest timepoint of 6 weeks. We do not consider either infection to be due to the cell-based implant, although both were likely induced or exacerbated by chronic immunosuppression. Pig 8 was successfully implanted with only 3 days of perioperative dexamethasone without long-term immunosuppression or corticosteroids.

### In vivo phonation

The method previously described for *in-vivo* canine phonation was successfully adapted here for Pig 4, at 6 months after COVR implantation [26,27]. For simplicity bilateral vagal nerve stimulation was employed. This produced vocal fold phonatory adduction. High-speed videolaryngoscopy recorded vibration and glottic closure; see S1 Video.

**Fig 7:**
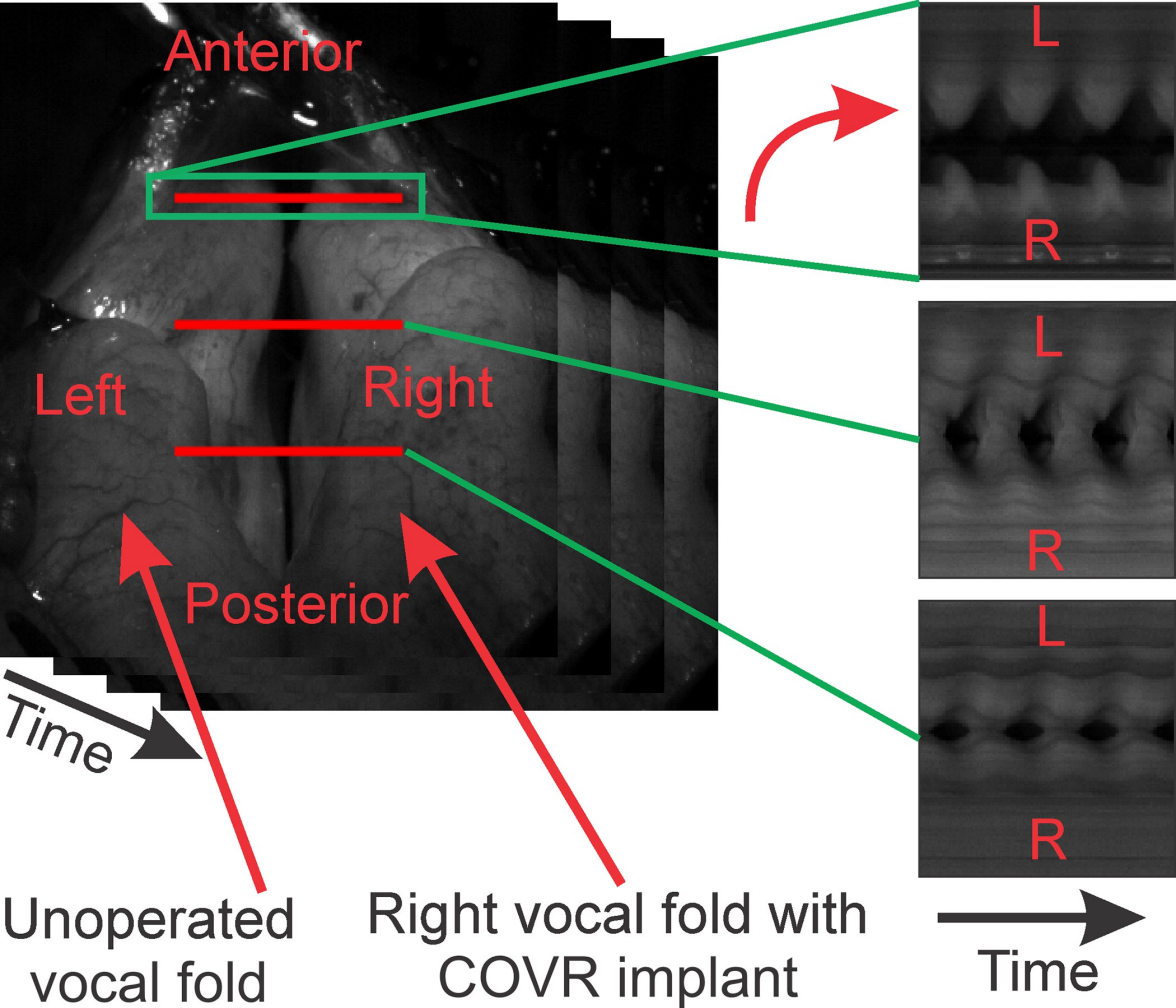
Creation of kymographs based on the high-speed video of pig 4 (6 months after COVR implant on right side) for three different anterior-posterior lines. Vocal folds maintained only a slight anterior glottic gap and otherwise mostly symmetric vibration as can be seen from the kymographs.

Vocal folds were assessed qualitatively using kymographs for illustration. As depicted in Fig 7, three anterior-posterior lines on the vocal fold were selected and kymographs, representations of changes in these lines over time as an image, were generated. Vocal folds maintained only a slight anterior glottic gap and otherwise mostly symmetric vibration.

### Microscopy

**Fig 8:**
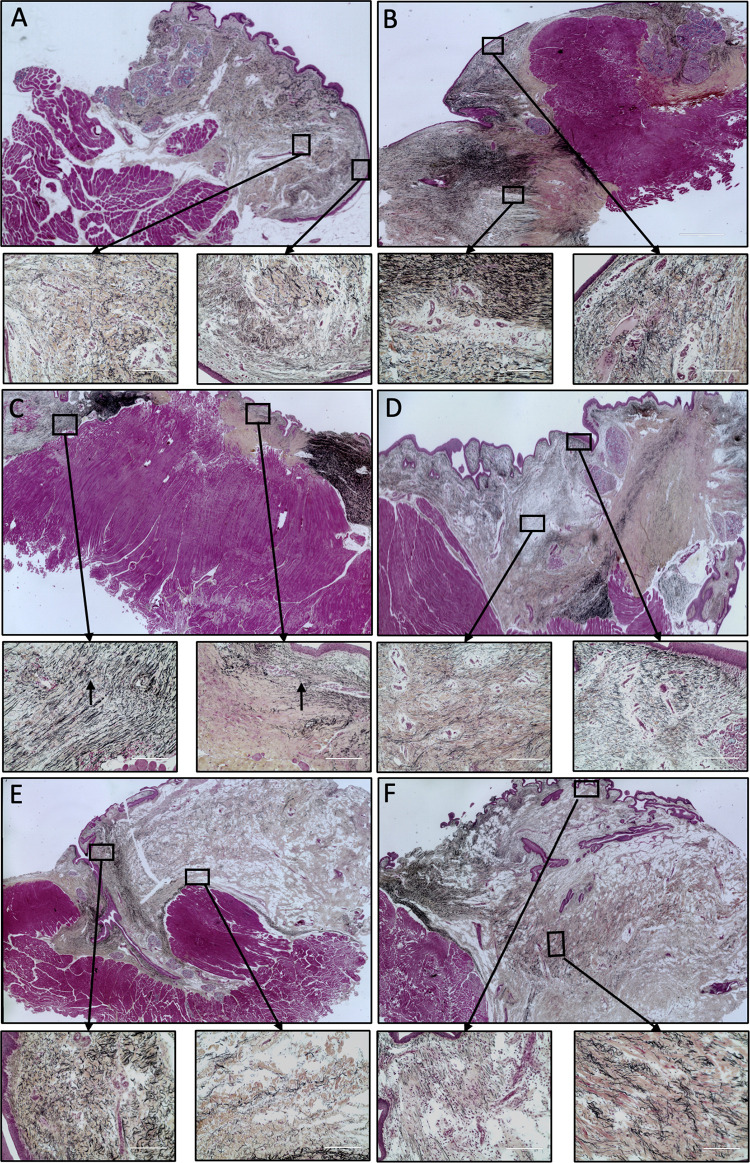
Movat’s pentachrome stain of porcine vocal folds 6 months after larynx surgery. Left column shows operated VF, and right column shows the contralateral unoperated VF for each (10X). Scale bars show 400 um. In all boxes, the epithelium and layers of the superficial and deep lamina propria are represented, with epithelium oriented at upper left, and thyroarytenoid muscle at lower right. A) hASC injection (pig 1); B) contralateral unoperated. C) Resected VF (scar, pig 2); D) contralateral unoperated. E) COVR implant (pig 4); F) contralateral unoperated.

Fig 8 shows histologic staining in pigs 1, 2 and 4 (only one vocal fold was injured, the other served as a control). Movat’s pentachrome demonstrates elastin (black), collagen (yellow) and glycosaminoglycans (blue). Thyroarytenoid muscle appears fuchsia in color. In pig 1 (hASC injection) and pig 4 (COVR), the lamina propria was reconstituted by a new tissue layer resembling the opposite unoperated side. In contrast, pig 2 (VF resection alone) showed marked thinning of lamina propria on the operated side, prominent yellow appearance of collagen, and clumped elastic fibers localized only to the surface layer. The injured pig 2 operated VF appearance was dramatically different than its contralateral control VF, whereas operated VF in pigs 1 and 4 that received treatments were more difficult to distinguish from contralateral controls.

**Fig 9:**
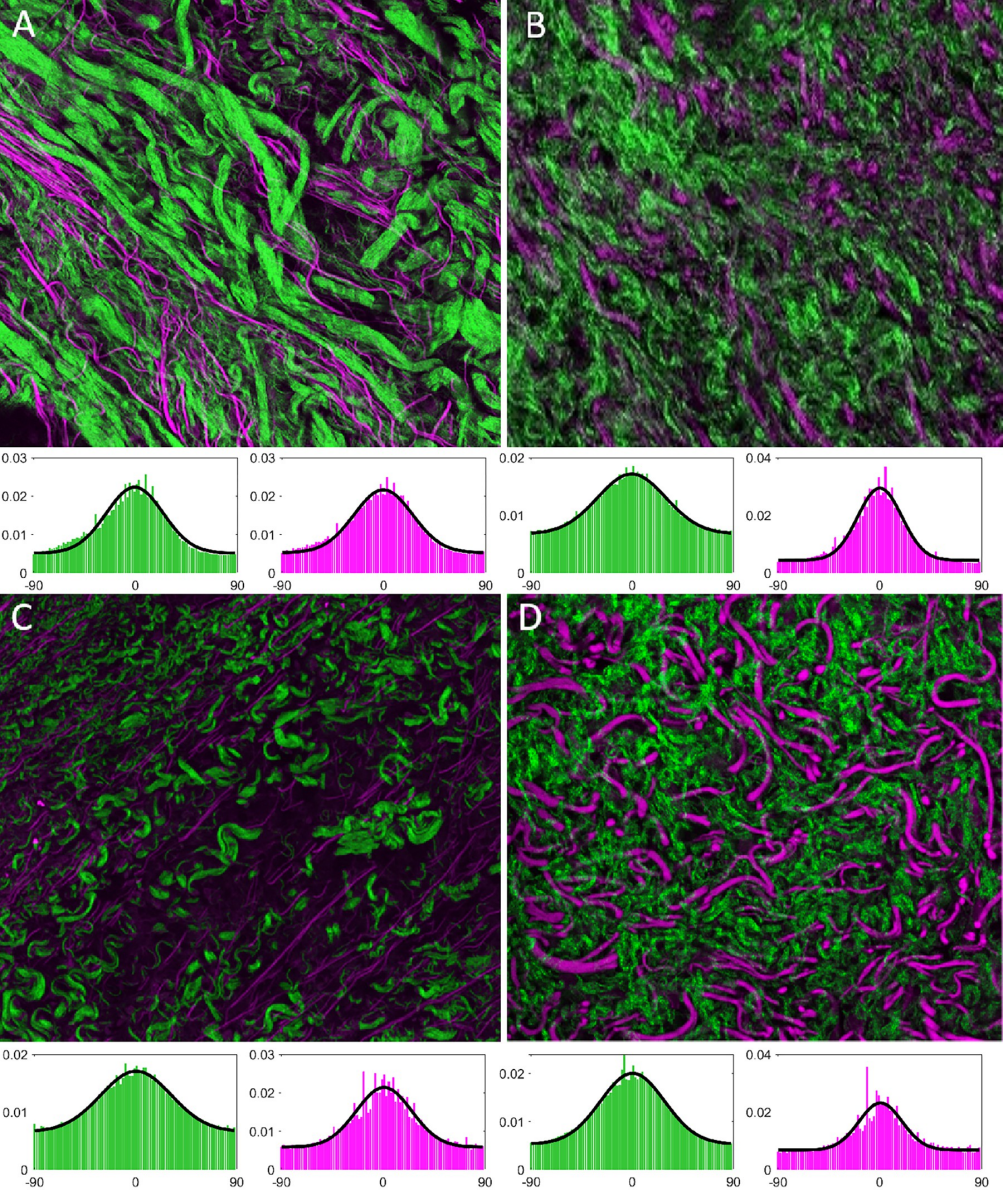
Nonlinear scanning laser microscopy of porcine vocal folds six months after surgery. Each image shows a single 40X field of view with collagen in green and elastic fibers in purple. Corresponding directionality distribution histograms and normal distribution fits for the entire lamina propria are shown below after centering the distributions at 0° (collagen in green and elastin in purple). A) Normal VF (unoperated, pig 4); B) hASC injection (pig 1); C) resected VF (scar, pig 2); D) COVR implant (pig 4).

Nonlinear scanning microscopy with Second harmonic generation (SHG) imaging demonstrated similar findings, with collagen and elastin present after surgery in pig 1 (hASC-injected) and 4 (COVR-implanted) (Fig 9). Pig 2 (VF resection) showed marked decrease in elastic fibers, and short wavy collagen. The COVR implant (pig 4) had the most prominent elastic fibers. Both collagen and elastin were shortened and without apparent directional alignment.

### Image-based analysis of fiber directionality and distribution

Mosaic images of the entire lamina propria from one slide from each animal (pigs 1, 2 and 4) were used for quantitative analyses of fiber directionality. A summary of all parameters is given in Table 3. Alignment differences were low in all cases, with less than a 10° difference between the peak alignments of collagen and elastin. This corresponds with the impression from Fig 9 that fibers generally run in parallel. A notable exception was the pig 4 COVR implant (Fig 9D) which shows less apparent directional alignment on visual inspection. Goodness of Fit is also lowest for that case (0.84 for elastin), reflecting less regular fiber distribution. Dispersion of collagen was greater in all operated VF compared to normal VF. This indicates greater collagen randomness after resection surgery compared to normal VF. The COVR implant condition did demonstrate the closest collagen dispersion value to normal VF. However, no statement can be made about statistical significance based on these observations.

**Table 3:** Parameters calculated from directionality distributions for collagen / elastin in all SHG images.

Measure	Pig 4, control	Pig 1, hASC	Pig 2, scar	Pig 4, COVR
Alignment difference (degrees)	4.7	9.7	8.1	3.2
GoF (a.u.)	0.95 / 0.96	0.95 / 0.96	0.95 / 0.90	0.97 / 0.84
Dispersion (a.u.)	25.1 / 25.8	30.4 / 19.5	31.4 / 24.5	28.5 / 19.1

### Amino acid quantitation

**Fig 10:**
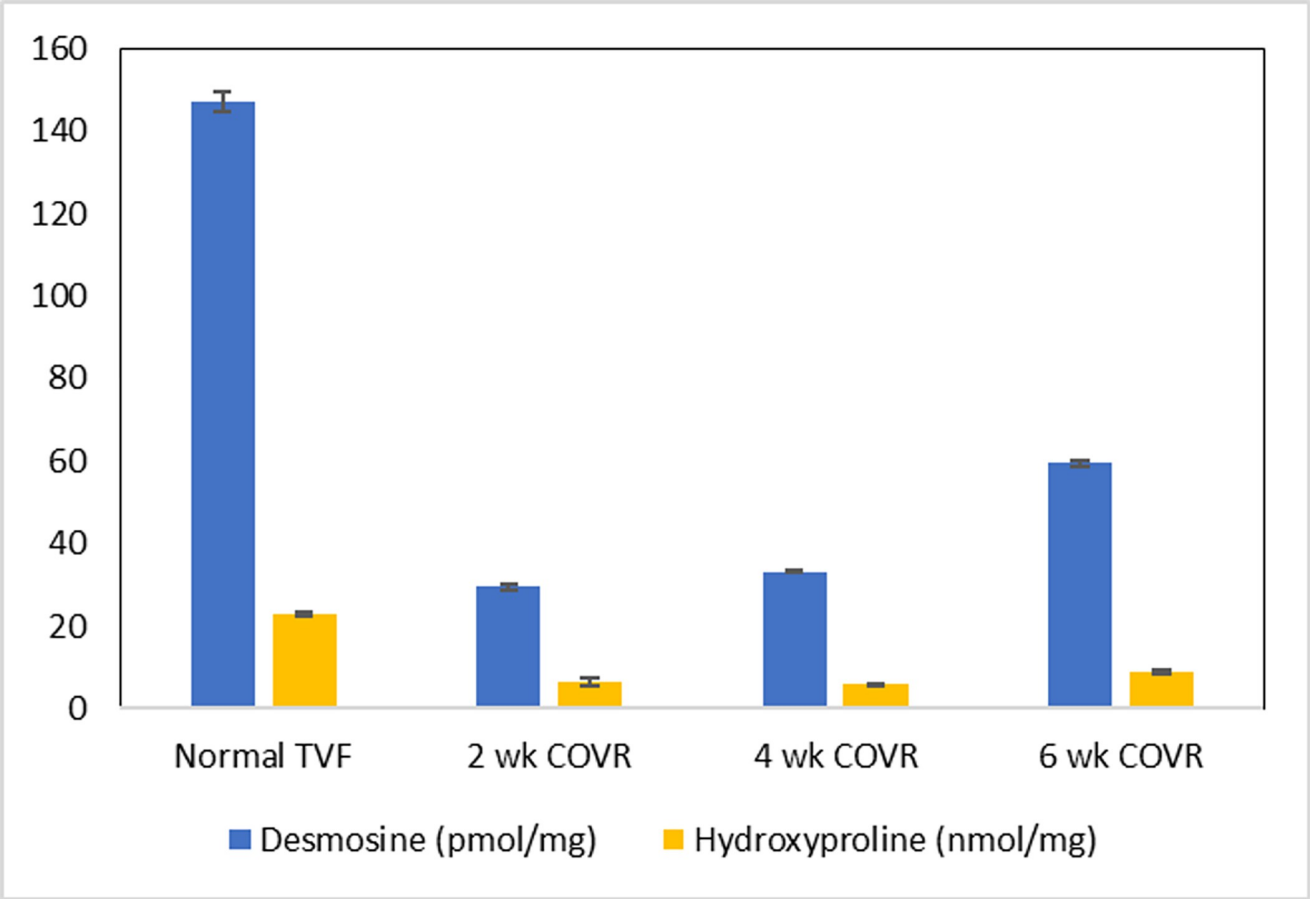
Amino acid quantitation. One porcine VF at each timepoint after COVR implant surgery was used for measurement of the amino acids desmosine/isodesmosine and 4-hydroxyproline by LC–MS. Quantities were normalized to dry weight of tissue. Error bars show standard deviation between replicate samples.

The amino acids 4-hydroxyproline (4HOP) (a component of collagen) and desmosine (D) (an amino acid present in elastin) were measured by LC-MS in one normal pig and in pigs 5, 6, and 7 undergoing short-term COVR implant (Fig 10). The amount reported for D includes equal co-elution with its structural isomer, iso-desmosine (isoD). Both 4HOP and D increased in the COVR animals over time, but neither reached the level in the normal animal during the 6-week post-operative period. Of note, D/isoD content increased at a more pronounced rate than 4HOP over the 6-week post-operative period, suggesting more rapid elastin deposition than collagen. Statistical analysis was not performed since each timepoint comprised only one sample.

### Acoustic parameter analysis

Parameter-average values and standard deviations are given in Table 4 for both evaluation A and B for pigs 3, 4 and 8. For evaluation A, Q50 and Flux1 displayed a statistically significant change (p<0.05) between pre-operative and early post-operative recordings in all three pigs, confirming the results from our previous publication [22]. For the more extensive evaluation B, Flux2, P60, and LPC8 displayed statistically significant changes between pre-surgical and immediate post-operative timepoints. Changes in Spread were not significant after correction for multiple testing. Overall correlations between the four parameters in evaluation B were low to negligible with correlations not exceeding 0.329 [35].

**Table 4:** Average values / standard deviations of acoustic samples recorded before and within 2 weeks after COVR implant surgery for P<0.05 for all parameters in all pigs except Spread which was not significant after correction.

	3 pre	3 post	4 pre	4 post	8 pre	8 post
Q50	2002 / 1831	1237 / 1042*	524 / 505	293 / 120*	763 / 469	173 / 69*
Flux1×10^3	7.53 / 4.98	7.26 / 2.78*	14.46 / 7.39	16.39 / 6.20*	5.69 / 0.84	15.22 / 3.42*
Flux2×10^1	4.11 / 1.29	5.02 / 1.25*	5.09 / 1.15	5.44 / 1.35*	X	X
Spread	1422 / 370	1412 / 244	1060 / 621	869 / 494	X	X
P60	69.13 / 36.94	55.09 / 29.89*	29.15 / 29.23	13.41 / 9.60*	X	X
LPC8	-1.18 / 1.28	-0.48 / 0.57*	-0.46 / 0.68	-0.16 / 0.26*	X	X

Longer acoustic followup revealed voice improvement beginning after about 2 months in pig 4. Statistically significant changes occurred in Flux1 when comparing 0–14 days versus 50–100 days after surgery, and in both Q50 and Flux1 when comparing 50–100 days after surgery with greater than 150 days after surgery. Both parameters returned to pre-surgery ranges in the long-term. For pig 3, Q50 decreased slightly over time and Flux1 increased, indicating a deterioration of acoustic features. This animal then succumbed to an infectious disease at post-operative day 40. Parameter values for pig 8 remained stable at abnormal levels throughout the 6-week observation period.

**Fig 11:**
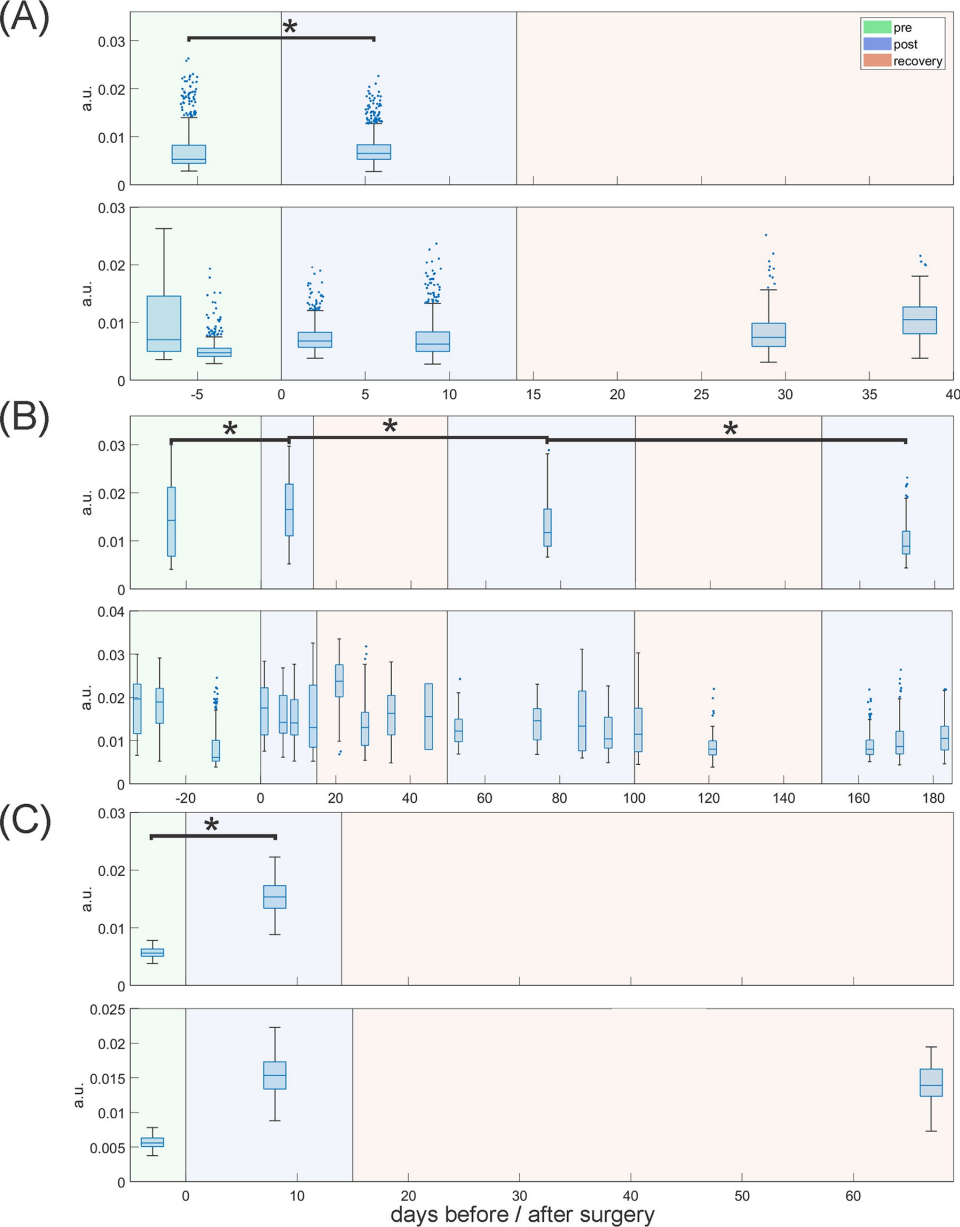
Change of Flux1 over time before and after COVR implant in (A) pig 3, (B) pig 4 and (C) pig 8. For statistical comparison in each section the top row shows boxplots of the random samples taken for analysis from the different time ranges (before surgery, up to 14 days after surgery, 50–100 days after surgery and more than 150 days after surgery). Bottom rows shows all recorded squeals on the dates indicated in the X-axis. The X-axis separates days before and after surgery at day 0. Data in the < 0 days and 0–14 days area was compared for pre-post-surgery comparisons. Pig 4 (B) also includes long-term comparisons. Asterisks indicate p<0.05.

**Fig 12:**
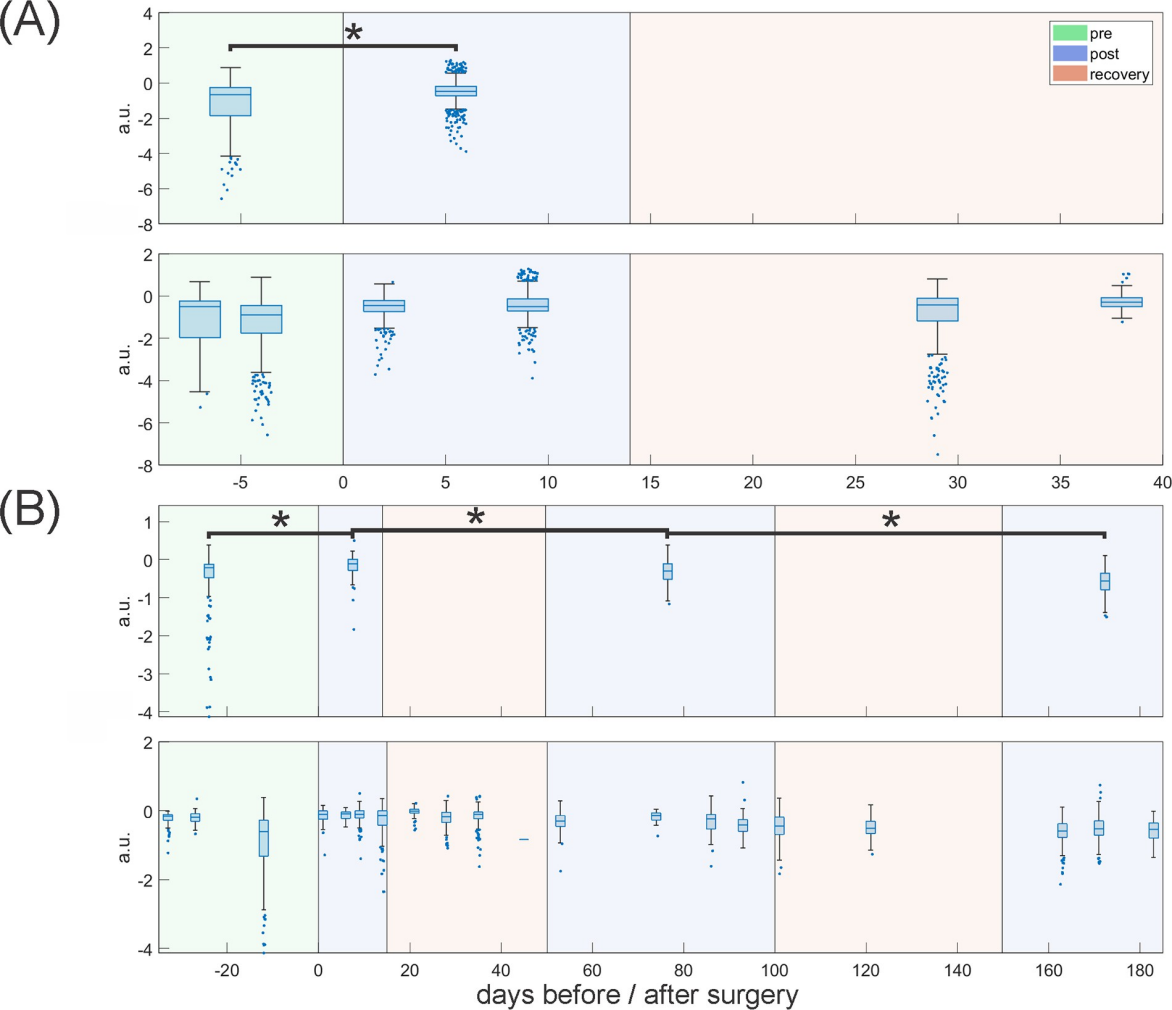
Change of LPC8 over time before and after COVR implant in (A) pig 3 and (B) pig 4. For statistical comparison in each section the top row shows boxplots of the random samples taken for analysis from the different time ranges (before surgery, up to 14 days after surgery, 50–100 days after surgery and more than 150 days after surgery). Bottom rows shows all recorded squeals on the dates indicated in the X-axis. The X-axis separates days before and after surgery at day 0. Data in the < 0 days and 0–14 days area was compared for pre-post-surgery comparisons. Pig 4 (B) also includes long-term comparisons. Asterisks indicate p<0.05.

Figs 11 and 12 show Flux1 and LPC8 values over time. The top row of each sub-figure shows the samples taken for statistical comparison among timepoints, and the bottom row shows all recorded squeals at their actual recording dates. Strong variation is apparent among different recording dates, even before surgery, and is indicative of the natural variability in spontaneous phonation. In general, Flux1 appears to deviate from pre-surgery levels over time in pig 3 (row 10-A), decrease to more normal levels in pig 4 (row 10-B) and remain abnormal through the limited 6-week observation period in pig 8 (row 10-C). Similar trends occurred for evaluation B parameters. LPC8 values remain consistently elevated for pig 3 up to 40 days, and begin to decrease after 2 months in pig 4. When comparing 0–14, 50–100, and 150+ days, the improvement of LPC8 was statistically significant and returned to preoperative levels over time. Other acoustic features were less consistent: Spread was significant when comparing 0–14 and 50–100 days, P60 was only significant comparing 50–100 and 150+ days, and Flux2 was not significant.

**Fig 13:**
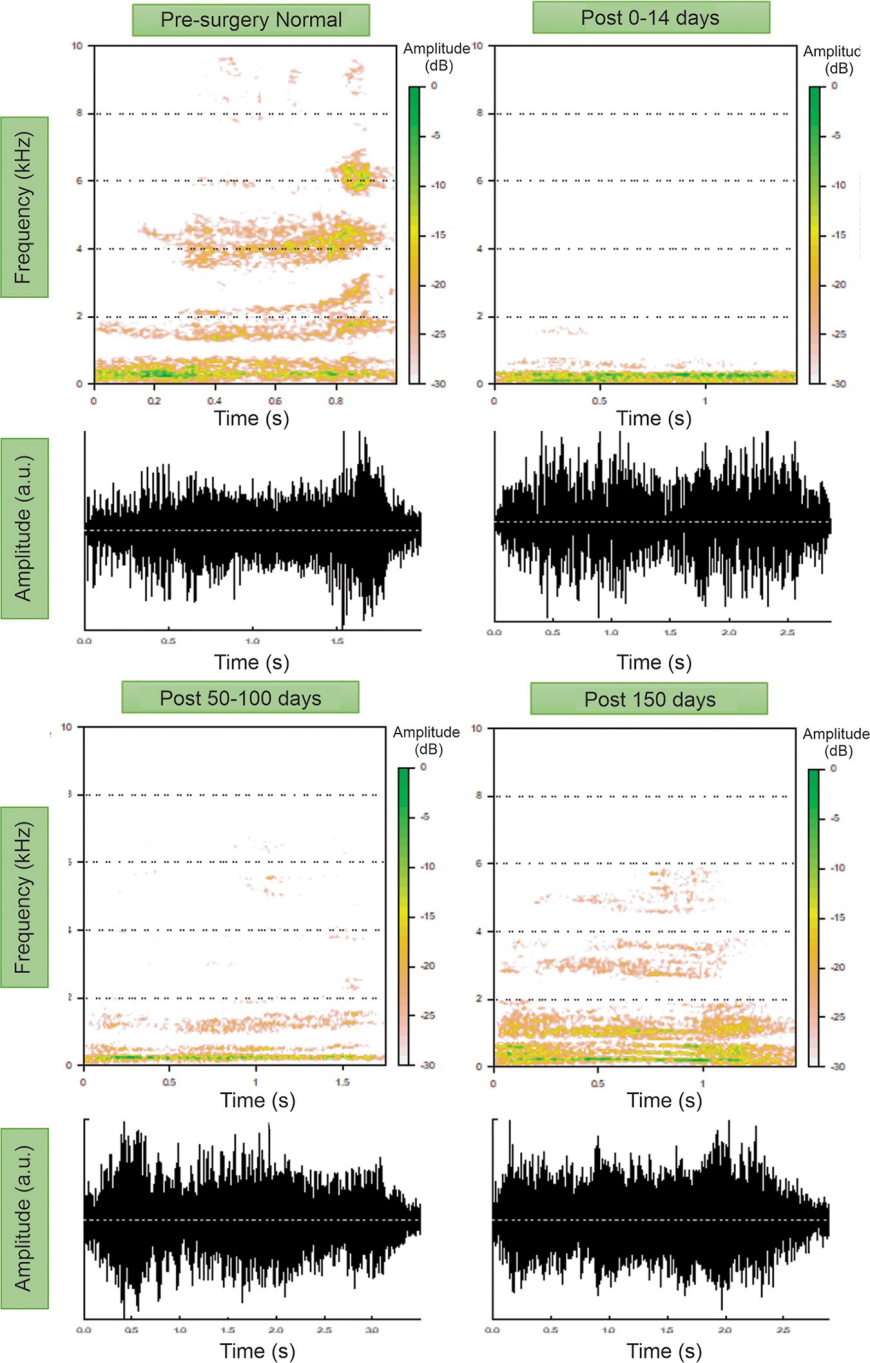
Example spectrograms from Pig 4 squeals recorded before COVR implant surgery, up to 14 days after surgery, between 50 and 100 days after surgery and more than 150 days after surgery.

To further illustrate the gradual recovery of pig 4, squeals from various time-points are shown in Fig 13. Real signal and spectrograms are depicted. Squeals were selected randomly within the time periods noted, and were verified to exhibit parameters close to median values. These examples show dramatic loss of high-frequency acoustics early after surgery, with gradual partial recovery.

## Discussion

In this pilot study we sought to develop methods for VF mucosal implantation and assessment in a voicing pig model. The purpose of the COVR is to allow for en bloc replacement of vocal fold mucosa and lamina propria, for use in cases of severe vocal fold scarring or tissue resection. In rabbits, we have shown that implanted COVR grafts recapitulate the layers of the native vocal fold [14,15]. However, rabbits’ lack of natural phonation limits their utility as a model for vocal recovery. Therefore in this study we chose Yucatan mini-pigs to study spontaneous vocal function and acoustic recovery in conjunction with structural analyses of the regenerating vocal fold. Unlike humans, objective analysis of porcine phonation has been limited. This study combines two independent acoustic analysis pathways to identify parameters that are meaningful for porcine voicing in the setting of laryngeal surgery.

### Surgical feasibility and safety

A surgical protocol for transcervical VF implantation of Yucatan mini-pigs was developed. This breed was chosen due to its ease of husbandry, with a maximum adult weight around 80–100 kg, and an average weight of 50 kg at 12 months [36]. In contrast, other swine breeds described previously for laryngeal surgeries, such as Landrace [19,21] and Yorkshire pigs, [37] continue to grow and can even achieve 200 kg over the 6–12 month intended implantation period [36]. Animals of that size are expensive to maintain and challenging to house and handle in an urban research university setting. The Yucatan breed poses a different challenge however, with a longer snout that complicates endoscopic laryngeal access. Our initial goal of performing endoscopic implantation (as is intended for humans) therefore had to be revised due to poor accessibility. During that surgical development, direct endoscopic injection of hASCs into exposed thyroarytenoid muscle after lamina propria resection was performed in one animal. This attempt was based on previous work that has demonstrated benefit from injection of hASCs directly into muscle [38,39]. We have also compared similar intramuscular hASC injection with COVR implant in rabbits, to determine if the cells alone can provide benefit [15]. The findings here from one pig were similar to our previous work in rabbits, with cell injection improving wound healing outcomes relative to cordectomy alone, but not achieving the degree of lamina propria restoration that COVR did. For further studies of three-dimensional implantation, the trancervical approach with midline pharyngotomy was found to provide well-tolerated and clear endolaryngeal access in Yucatan mini-pigs.

Results from this pilot study highlight that long-term immunosuppression might not be necessary after laryngeal hASC administration. An immunosuppression regime comprised of long-term cyclosporine and prednisolone was trialed in our study and eventually revised to prednisolone alone after a meningitic infection in pig 3, and rejected altogether after pig 7 exhibited an endemic porcine bacterial illness. Ultimately, the final animal in our study, pig 8, was implanted with the COVR implant with only 3 days of perioperative corticosteroids for the purpose of reducing laryngeal edema. Pig 8 exhibited no adverse events and survived to the planned timepoint of six weeks with no clinical evidence of transplant rejection despite its competent immune system. Although rejection by the recipient’s immune system can be a barrier to cell-based therapies in life-sustaining organs [40,41], the field of regenerative medicine is moving away from the use of long-term immunosuppression. Rather, most therapies now have a goal that transplanted cells should eventually be replaced by host cells, or form a stable chimera with immune tolerance. Fortunately, MSCs in particular actively suppress the immunologic responses that cause rejection of most transplanted tissues [42–45]. Such local immunosuppressive effects from the hASCs in the COVR implant may have contributed to immune tolerance in pig 8 and in our previous rabbit studies. As such, we plan to not administer immunosuppression in future studies. Additionally, we observed that the two animals who became ill were smaller and younger at initial surgery, so we now recommend a minimum weight of 30 kg to reduce risk of medical complications.

### Structural regeneration

Wound healing was studied here at early (2–6 weeks) and late (6 months) timepoints. The early timepoints were chosen to capture the rapid remodeling phase with collagen deposition, while the late timepoint should better reflect longer-term matrix remodeling and better capture elastic fiber deposition. In the early phase, amino acid markers for collagen and elastin were quantified after COVR implantation. Both were significantly reduced at 2 weeks postop compared to normal, but began to recover at 4–6 weeks indicating deposition *in vivo*. Longer timepoints and more replicates are warranted.

The microscopic evaluations presented here demonstrate findings at the later timepoint of six months. The vocal folds receiving either hASC injection or COVR implant had substantially less collagen than the resected (scarred) VF. Notably, elastin content appeared increased in the hASC condition and further increased in the COVR condition, which is expected to improve vibratory properties. These findings are limited by their qualitative nature and the n = 1 in each case.

Nonlinear scanning microscopy with second harmonic generation (SHG) was explored as an objective, quantifiable method to identify and characterize collagen and elastin fibers. SHG detects these molecules based on their characteristic light emitting properties following wavelength-specific excitation, thus avoiding the ambiguity that arises with traditional histologic or immunohistochemical techniques. The single-channel images are amenable to quantitative image analysis. In the SHG images studied here, all operations were found to produce broader distributions of collagen alignment (more randomness) than normal VF; COVR was the closest to normal. Qualitatively, the most elastic fibers were present in the COVR (among operated VF), but they did not appear strongly aligned. It is possible that elastic fiber alignment would increase with longer recovery times *in vivo*, consistent with the natural postnatal development of human vocal folds which takes months or years [46,47]. Alternatively, supplying directionality cues during the *in vitro* COVR preparation may improve its ultimate fiber alignment *in vivo*.

The COVR implant concept studied here is designed to provide a resorbable fibrous template for subsequent ECM remodeling and host cell ingrowth. It may be advantageous over acellular or injectable scaffolds in that the implant contains a bilayer of cells within a fibrous structure that better matches the native vocal fold. The regenerating tissue can benefit from molecular signals between the epithelial and mesenchymal cells, and between the implanted cells and host tissue. Mechanistic questions regarding how these signals reduce subsequent fibrosis are not the focus of the current study, however, and are better studied in a smaller animal model such as rabbits. In previous work, MSCs have been found to have anti-inflammatory properties and to reduce disordered collagen deposition when injected into scar [39,44,48]. Consequently, this results in improved viscoelastic properties, vibrational characteristics, and vocal function over time [10,49]. Questions remain regarding the ideal clinical criteria for injections alone, such as suitability for repairing severe scar or for acute reconstruction at the time of cordectomy. Here, one animal was treated solely with hASC injection at the time of acute resection, and qualitative and quantitative results suggested intermediate results between injured control and COVR implant at 6 months.

### Acoustic recovery

Assessing vocal fold function in any voicing mammal requires acoustic analyses. Yet, even for humans, there is little consensus about how to define a “normal” voice and objective measures to define improvement after a disease or surgery vary widely. The matter becomes more complicated when dealing with animals that cannot be instructed to phonate on command. The use of parameters designed for humans is ill-advised, as pig phonation is characterized by nonlinear behavior and chaotic qualities without subharmonics, harmonics, or complex nervous system control [50,51]. This means that more established voice measures reliant on harmonic structure such as Jitter or Cepstral peak prominence should not be used in pig phonation [22].

We recently identified objective measures for porcine phonation that are sensitive to laryngeal surgery [22]. As in that initial work, this study investigated only high-pitched pig phonation events, generally referred to as “squeals”. These are thought to originate from the animal’s vocal folds [50] in contrast to lower-pitched phonation such as grunts that more likely originate from the non-laryngeal vocal tract [52]. We avoid a more specific differentiation, as there are no unanimous rules to separate call types [23–25,52,53]. The blurring between call types [24,25] and extensive variation in normal pig calls [52,53] demands a more general definition and a larger sample size of extracted squeals is preferable over a narrower definition that also may be harder to delineate and replicate.

For this study, we used a larger set of squeals to both validate the previously identified parameters and to define additional parameters for vocal recovery in pigs. Two separate evaluations (which we refer to as A and B) were found to be generally in good agreement in the three investigated animals. The parameters identified as statistically significant in the two independent acoustic analyses are similar. Conceptually, the P60 parameter is similar to Q50 (both are spectrum markers), and Flux2 is similar to Flux1 (both are different implementations of the same parameter). The fact that two independent analysis approaches resulted in the selection of similar parameters validates the hypothesis that the features captured by these parameters decisively identify changes stemming from laryngeal surgery (i.e., changes in high frequency harmonics). The only acoustic parameter presented that did not differ significantly pre- and post-surgery was Spread, with p-values between 0.1 and 0.2 after Bonferroni correction. However, since the Bonferroni correction is conservative, and Spread had low correlation with the other selected measures, it was decided to include it in the final parameter set for evaluation B. Spread could still be of value in assessing pig health, especially in datasets with less variability between squeals.

Regarding acoustic recovery, the acoustic changes observed post-operatively reversed towards normal about two months after COVR implant in the one available animal. The objective findings from parameter analysis correlate with the more subjective perceptual assessment and examination of spectrograms. This consistency between parameter analysis and perceptual assessment further supports the validity of the parameters identified to capture critical porcine acoustic features. A larger, controlled study including injured but not implanted pigs is required to assess the reproducibility of acoustic recovery.

The parameters studied also reflected overall health status. Pig 3 contracted meningitis during the experiment, and its condition was reflected in its vocal parameter values. All investigated parameters from evaluation A and evaluation B did not show recovery for this individual. On the contrary, values appear to temporally deteriorate as was the case for Flux1 depicted in Fig 11. Pig 4, on the other hand, recovered well after surgery and the acoustic parameters reflect this by moving toward pre-surgery values (Figs 11 and 12). Nevertheless, parameter values all show considerable variation within and between recording dates which made it difficult to draw definitive conclusions. Pig 8 showed a clear change in parameter values before and after surgery but no subsequent improvement. However, this animal had fewer recorded squeals, fewer recording dates, and shorter follow-up than the other pigs. Therefore, observed trends are less substantiated than for the other pigs, despite generally more consistent data. In general, we conclude that acoustic measures in all investigated animals reflect expected voice changes, with short-term worsening of voice quality after surgery, further worsening with overall health condition (pig 3), and gradual improvement and recovery over several months (pig 4).

### Limitations

This needed study was undertaken as a pilot project to refine methods in a new animal model and explore a new approach to evaluate porcine acoustic data. Only one pig underwent long-term implantation, thus limiting the conclusions that can be drawn. One injured control pig was included, but only post-operative acoustic data were recorded. As was subsequently discovered, there is large variation between pigs’ inherent voice qualities. Therefore, we were forced to interpret each pig’s acoustic data in relation to that same individual’s pre-operative voice, using each subject as its own control. Further, for acoustic analysis, no long-term control was available to assess the possibility of spontaneous recovery after surgery without subsequent COVR implantation. In light of these limitations, conclusions cannot be made regarding efficacy of the COVR therapy based on these data. A larger study is planned using the methods developed here, with more pigs and the inclusion of injured controls with full pre- and post-surgery acoustics.

Human-derived ASCs were chosen in order to best predict the ultimate behavior of a human implant. One caveat of using xenograft hASCs rather than pig ASCs is the potential for an adverse immune response to the implanted cells. However, ASCs are known to have immunosuppressive properties which may partially protect them from rejection [40]. We have reported partial cell persistence up to 4–6 weeks in rabbits receiving COVR implants with either allogeneic rabbit ASC or xenogeneic human ASC without immunosuppression [15,16]. Host cells migrated into the new tissue to restore long-term function. We therefore hypothesize that permanent engraftment is not required in the vocal fold implant, with the hASC action being most critical during initial wound healing. It is also important to note that the immunosuppression protocol changed throughout the series of 8 animals, which limits the reliability of direct comparisons. Lessened immunosuppression, first in pigs 4–7 which received only prednisolone, then in pig 8 which had no long-term medications, raises the likelihood of immune response towards the implanted cells. Nonetheless, pig 8 exhibited no adverse health events during its 6 weeks of follow-up after immunocompetent xenotransplant. Therefore, we plan for future animals to undergo hASC-based COVR implantation without immunosuppressive medications.

## Conclusion

This study investigated structural and functional changes that emerged in eight pigs after vocal fold surgery implanting a Cell-based Outer Vocal fold Replacement. Important methodological refinements included adoption of a transcervical surgical approach for these Yucatan mini-pigs, and elimination of immunosuppression. An *in vivo* phonation procedure was developed and demonstrated. Long-term microscopy data in one animal per group suggested that the COVR implant better recapitulated native vocal fold structure than hASC injection or healing by secondary intention after resection. Ex vivo measurement of the amino acids desmosine /isodesmosine and 4-hydroxyproline in excised tissues provided a quantitative and sensitive measure of early tissue recovery following surgery and implantation. For functional analysis, spontaneous phonations from three pigs with COVR implant were evaluated, recorded up to one month before and up to six months after surgery. Parameters Q50 and Flux1 based on one previous study [22] as well as parameters Flux2, Spread, P60, and LPC8 were calculated for all extracted squeals. These parameters in general reflected pig health and voice recovery after surgery, with vocal improvement beginning around 2 months after COVR implantation. The acoustic parameters also reflected the inherent variability of spontaneous voicing, the capacity for which we aim to restore after vocal fold surgery.

## Supporting information

S1 Table Directions of tests along with parameter names, units, short descriptions and sources for the final set of six parameters. https://doi.org/10.5281/zenodo.7783488.
(DOCX)

S1 Video Demonstration video of *in-vivo* pig larynx phonation. (MP4)
