# Supplementary material for: Tissue-engineered vocal fold replacement in swine: Methods for functional and structural analysis
Source: PLoS One. 2023 Apr 21;18(4):e0284135. doi: 10.1371/journal.pone.0284135 (PMC10120936; doi:10.1371/journal.pone.0284135)
Supplement: S1 Table — https://doi.org/10.5281/zenodo.7783488. (DOCX) [file pone.0284135.s001.docx]

**Table S1.** Parameter abbreviations, expected change from pre- to post-surgery and a short explanation of the parameter and reasoning behind this hypothesis.

| **Parameter, unit** | **H1** | **Reasoning** | **Source** |
| --- | --- | --- | --- |
| Q50, Hz | decreases | Q50 is the 50% energy quantile in the energy spectrum. The share of high frequencies decreases after surgery. Therefore we also expect a decrease of Q50 as respectively more of the total energy is contained in lower frequencies. | [22] |
| Flux_1_, a.u. | increases | Flux measures the rate of change of the frequency spectrum. Squeals may become more noisy and chaotic after surgery, therefore we expect the change in energy between signal windows and therefore Flux to increase. | [22] |
| Flux_2_, a.u. | increases |  | [29] |
| Spread, Hz | decreases | Spread (spectral) is the standard deviation around the spectral centroid. As the energy of the spectrum gets more concentrated around lower frequencies we expect Spread to decrease. | [30] |
| P60, Mel | decreases | Index of the Mel spectrogram bin to contain 60% of the total power. Analogously to q50 we expect P60 to decrease as a higher relative share of energy is expected in lower frequencies after surgery. | [31] |
| LPC_8_, a.u. | increases | Linear Predictive Coding coefficients of order 16 (lpc1-16). Linear Predictive Coding is typically used to analyze the speech signal by estimating the formants produced by the vocal tract. Hence any major changes in the vocal tract will mostly result in changes in the LPC coefficients. Based on observations on initial LPC measures it is expected to increase. | [32] |
